# “*Currently flying blind*” Stakeholders’ perceptions of implementing statewide population-based cancer staging at diagnosis into the Western Australian Cancer Registry: a rapid qualitative process evaluation of the WA Cancer Staging Project

**DOI:** 10.1186/s12913-023-09662-7

**Published:** 2023-07-15

**Authors:** Stephanie Smith, Richard W. Trevithick, James Smith, Li Pung, Karen Taylor, Ninh Ha, Kevin E. K. Chai, Cristiana Garcia Gewerc, Rachael Moorin

**Affiliations:** 1 School of Population Health, Curtin University, Perth, WA Australia; 2 Curtin Medical School, Curtin University, Perth, WA Australia; 3 Department of Health, Clinical Excellence Division, Western Australian Cancer Registry, Perth, WA Australia; 4 Cancer Network WA, North Metropolitan Health Service, Nedlands, WA Australia; 5 School of Population and Global Health, The University of Western Australia, Nedlands, WA Australia

**Keywords:** Population-based cancer staging, Cancer registry, Cancer staging at diagnosis, Process evaluation, Qualitative

## Abstract

**Background:**

Cancer stage at diagnosis is essential for understanding cancer outcomes, guiding cancer control activities and healthcare services, and enabling benchmarking nationally and internationally. Yet, most cancer registries in Australia do not routinely collect this data. This study explored key stakeholders’ perceptions of implementing cancer staging utilising Natural Language Processing and Machine Learning algorithms within the Western Australian Cancer Registry.

**Methods:**

Perceptions of key breast and colorectal cancer stakeholders, including registry staff, clinicians, consumers, data scientists, biostatisticians, data management, healthcare staff, and health researchers, were collected. Prospective and retrospective qualitative proformas at two-time points of the Western Australian Cancer Staging Project were employed. The Consolidated Framework for Implementation Research was used to guide data collection, analysis and interpretation embedded in a Participatory Action Research approach. Data analysis also incorporated Framework Analysis and an adapted version of grading qualitative data using a visual *traffic light labelling system* to highlight the levels of positivity, negativity, and implementation concern.

**Results:**

Twenty-nine pre-proformas and 18 post-proformas were completed online via REDCap. The grading and visual presentation of barriers and enablers aided interpretation and reviewing predicted intervention outcomes. Of the selected constructs, complexity (the perceived difficulty of the intervention) was the strongest barrier and tension for change (the situation needing change) was the strongest enabler. Implementing cancer staging into the Western Australian Cancer Registry was considered vital. Benefits included improved knowledge and understanding of various outcomes (e.g., treatment received as per Optimum Care Pathways) and benchmarking. Barriers included compatibility issues with current systems/workflows, departmental/higher managerial support, and future sustainment.

**Conclusions:**

The findings aid further review of data gaps, additional cancer streams, standardising cancer staging and future improvements. The study offers an adapted version of a rapid qualitative data collection and analytic approach for establishing barriers and enablers. The findings may also assist other population-based cancer registries considering collecting cancer stage at diagnosis.

**Supplementary Information:**

The online version contains supplementary material available at 10.1186/s12913-023-09662-7.

## Introduction

There are over 1 million people living or who have lived with cancer in Australia [1]. In 2022, it was estimated that there were 162,000 new cases of cancer diagnosed and 50,000 deaths in Australia [2]. Findings from the Australian Burden of Disease Study showed that cancer as a disease group was the leading cause of burden in Australia in 2018, accounting for 18% of the total disease burden and 34% of the fatal burden [2, 3]. Within Western Australia (WA), there were 13,361 new cancer diagnoses, and over 4,147 people died from cancer in 2017 [4]. As the population in WA rises along with an ageing population, the number of diagnoses will continue to increase [5]. This highlights the need to better understand the population dynamics of cancer over time for healthcare systems [6].

Since 1982, the Western Australian Cancer Registry (WACR) has provided population-based cancer data for planning healthcare services and supporting cancer-related research at local, national and international levels [7]. Under the Health (Western Australian Cancer Register) Regulations 2011, health practitioners (e.g., pathologists, haematologists, and radiation oncologists) in WA have a legal requirement to notify the WACR of a malignant neoplasm within 30 days [4]. By this mandate, the WACR receives pathology reports, hospital morbidity data, and death notifications associated with cancer [8]. The WACR routinely collects data on tumour site, morphological type, date of diagnosis, basis of diagnosis, and additional demographic information [8]. This information is then used to monitor the number of cases of cancer in WA; plan, monitor, and evaluate services for the control of cancer and the care of cancer patients in WA; compile and publish general or statistical information relating to cancer; and carry out research into the causes, prevention, screening and treatment of cancer [4].

Cancer staging is an approach to classify the disease according to its extent and spread at the time of diagnosis [9] based on evidence acquired before treatment [10]. Staging data at the population level is essential for understanding cancer outcomes and guiding cancer control activities in population-based studies [11]. Staging information allows for a more complete analysis of trends and a thorough understanding of potential causal factors for underlying shifts in incidence and mortality over time [6]. Access to this information is invaluable to population health, including evaluating health inequalities [12] and access to and the impact of cancer screening, cancer-related healthcare interventions and services within and across jurisdictions [6] and countries [13].

The lack of national standardised collection of staging data in Australia is well known and was identified by Cancer Australia and addressed in the National Cancer Data Strategy [14]. This resulted in the subsequent funding of the Stage, Treatment and Recurrence (STaR) project in 2015 to collect registry-derived stage for the five high-incidence tumour groups (prostate, breast, lung, colorectal and melanoma) [14, 15] cross-sectionally for those diagnosed in 2011. All Australian State and Territory population-based cancer registries participated [16]. This data collection remains the only Australian population-based cancer staging data available and is already outdated. Registry-derived stage is a term created through the STaR project. It is defined as the best estimate of cancer stage at diagnosis derived from the available data sources used by population-based cancer registries [14]. For the STaR project, the Victorian Cancer Registry, in consultation with Cancer Australia, developed business rules. Registry-derived stage reflected a simplified stage classification system based on the commonly used classification system, the American Joint Committee for Cancer (AJCC) Tumour Node Metastasis (TNM) 7^th^ version [11], to enable the standardised collection of registry-derived staging across all state and territory cancer registries in Australia [14]. Business rules are a set of detailed instructions which outline the data components and decision-making process required for defining each stage category [17]. However, only the Victorian cancer registry has continued with the routine collection of cancer staging in Australia.

Since 2018, cancer stage has been opportunistically recorded in the WACR while coding routine data items from available data sources. It is not routinely collected and continues to be underreported, as previously noted [18]. More recently, the WA Cancer Plan 2020–2025 priorities for implementation identified the key strategic action to ‘Develop a timely data collection for cancer stage at diagnosis’ [19]. Considering that collecting stage at diagnosis is an additional data item in a setting of increasing cancer notifications in most cancer registries [6], further review of timely approaches is necessary. Therefore, there is a need to expand population-based cancer registries in Australia to include stage at diagnosis [20] and explore rapid, efficient and sustainable methods to access these data.

In recognition of these limitations, the WA Cancer Staging Project was initiated in June 2021, initially for 12 months, as a collaboration between Curtin University and the WACR. The following will be used interchangeably: WA Cancer Staging Project/the project and cancer staging at diagnosis/cancer staging. The project’s main aim is to develop and deliver statewide population-based stage at diagnosis for breast and colorectal cancer within the WACR, utilising Natural Language Processing (NLP) and Machine Learning (ML) for timely data collection. The project’s outcome is to have breast and colorectal cancer staging as business as usual, being routinely collected in the WACR. Not only is the use of NLP/ML a rapid approach, but in the recent rapid scoping review on determining cancer stage at diagnosis in population-based cancer registries, the benefits of using computer algorithms included a reduction in potential human error and variation compared to manual staging [21].

The use of process evaluation, defined as the evaluation of “individual, collective or management perceptions and actions in implementing any intervention and their influence on the overall result of the intervention” [22], focuses not only on the overall outcomes but also the specific intervention processes. It helps to understand the planning process and explains how and why decisions are made [23]. The focus includes the experiences and perceptions of the individuals involved [24]. As the project’s stakeholders are key to the project’s progress and direction and have a variety of expertise in cancer staging, it was essential to capture their perceptions. This qualitative process evaluation aimed to provide insight into the implementation of population-based cancer staging into the WACR and understand the delivery, functioning, impact and contextual factors from stakeholders involved in the WA Cancer Staging Project. The study was conducted towards the start and end of the first year of the WA Cancer Staging Project.

## Method and analyses

### The WA Cancer Staging Project Context

**Fig. 1:**
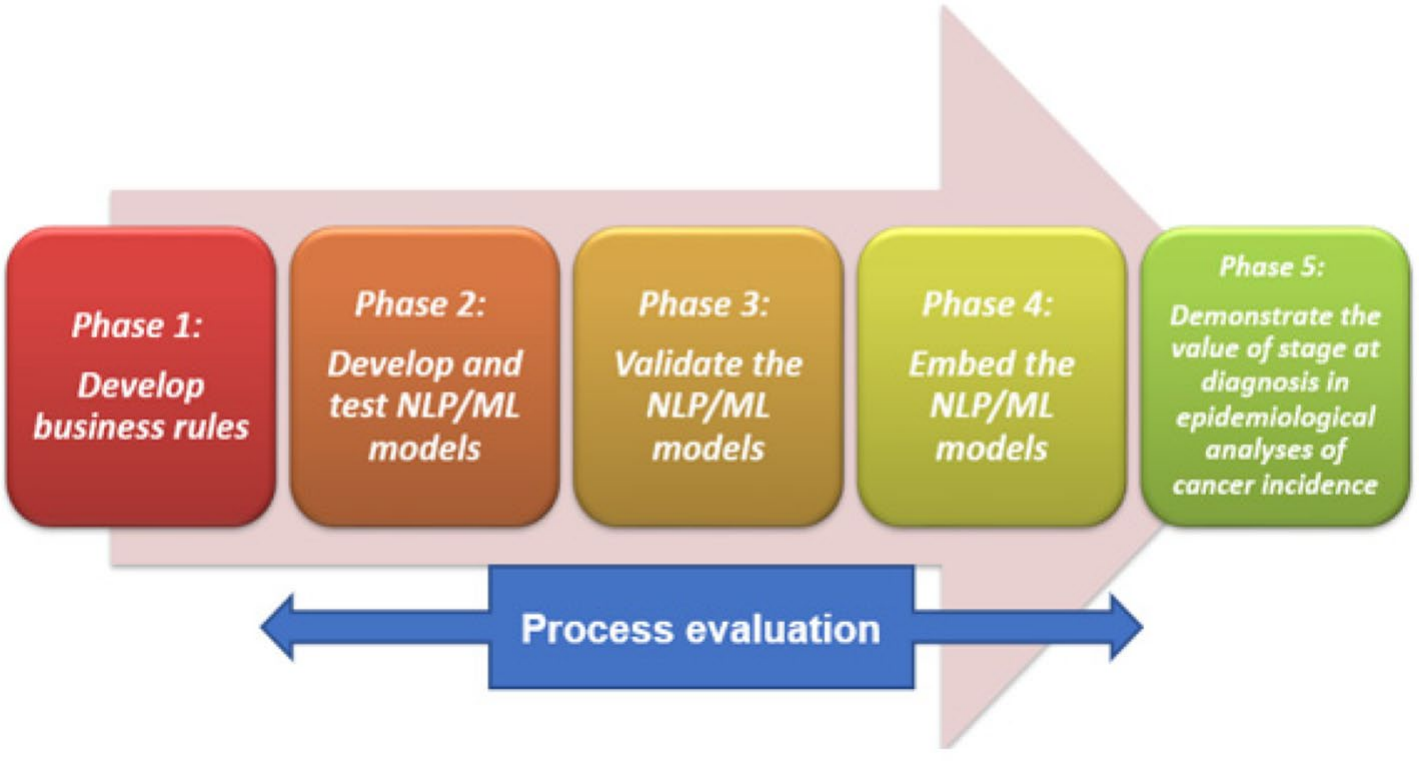
Phases of the WA Cancer Staging Project and implementation process

Project staff (Project Manager/Research Fellow, Project Officer/Research Officer, Data Scientists and Biostatistician) were recruited and started on the project between October 2021 and February 2022. The Project comprises five phases co-designed with key stakeholders involved in the project to develop critical foundation work in cancer staging to enable national and international benchmarking. Figure 1 outlines the five phases of the project’s implementation process. Table 1 provides an overview of the project and details each implementation phase. Breast cancer was selected due to having high incidence in WA [25]. Colorectal cancer was chosen due to additional complexities, including multiple primary tumours reported on pathology reports. Both tumour groups are screened for in national population-based screening programs [26]. Similarly, there is access and availability to data in the WACR for both tumour groups. Both were previously identified as tumour groups that could be staged with adequate completeness [16].

**Table 1:** Overview and descriptions of the WA Cancer Staging Project Phases

***Phase 1: Develop business rules.*** A Project Advisory Group (PAG) (to oversee the project and Working Groups) and Breast and Colorectal Cancer Working Groups (to consult on developing the business rules) were recruited to consult throughout the project. A snowball recruitment strategy to ensure a variety of expertise was used. The stakeholders were notified through team meetings about the project. The Project Manager/Research Fellow (SS), experienced in stakeholder engagement and was in a neutral position, invited potential stakeholders and potential stakeholders were advised who had recommended them. Invites to the PAG and Working Groups included the option to recommend a suitable stakeholder if the invitee was unavailable. The consumer representatives agreed that consumer input was more valuable in the PAG rather than the development of the business rules and data collection challenges that the Working Groups would oversee. Consumer representatives were still involved in overseeing the Working Groups and consulted on issues as required.
The findings from the rapid scoping review on determining population-based cancer stage at diagnosis in population-based cancer registries [21], which outlines various classification systems, were reviewed. Based on the evidence, the PAG advised using the AJCC TNM classification for cancer staging as it is the most used, established and adaptable system compared to other classification systems. The Victorian Cancer Registry’s business rules (based on a simplified version of the AJCC TNM, version 7 [11]) were compared against the full version [27] and the updated version 8 of the AJCC TNM [28] by the Breast and Colorectal Working Groups in the breast and colorectal business rules development. During this consultation, the PAG endorsed a **Cancer Staging Tiered Framework** for collecting stage data within the WACR to enable stage collection using current data sources and to ensure future proofing. The Staging Tiered Framework includes three tiers, 1) complete AJCC TNM stage, 2) Registry-derived stage, and 3) pathology stage. The WA Cancer Staging Project operates on the registry-derived tier based on currently available data sources.
***Phase 2: Develop and test NLP/ML models.*** To (i) classify cancer reports received according to cancer type to prioritise work to achieve timely cancer staging, (ii) enable automated extraction of information relevant to staging from pathology reports for breast and colorectal cancers and provide stage at diagnosis based on the business rules. The models were developed once the business rules were created (through the consultation with the PAG and Working Groups) to automate and support extracting information from relevant data sources to minimise or eliminate manual intervention. The development and testing of the models included classifying pathology reports into cancer types (e.g., colorectal and breast) and report types (e.g., biopsy, colectomy) in addition to extracting Tumour Node Metastasis (TNM) staging and related information.
***Phase 3: Validate the NLP/ML models.*** Validate the models against (i) manually coded data by the coding team, (ii) manually staged data (iii) clinically staged data. The models developed were trained on manually staged data from 2018 and 2019 and were validated on data from 2020 within the WACR. The validation on clinically staged data did not occur as data were received after the evaluation.
***Phase 4: Embed the NLP/ML models.*** Enabling future routine collection prospectively and retrospectively. A server was purchased to embed the models with the WACR server. The embedding of the models did not take place within the current study due to global shortages in receiving the purchased server for routine coding and analysis workflows. It was planned that the deployment of the new WACR analytics service was to retrieve reports from the WACR and Hospital Morbidity Data Collection (HMDC) databases and record the model outputs in the WACR database for monitoring and analysis.
***Phase 5: Demonstrate the value of stage at diagnosis in epidemiological analyses of cancer incidence.*** Perform epidemiological analysis of breast and colorectal cancer incidence in 2019 and 2020 using the staged data produced by the models according to demographic and other tumour-specific information routinely available within the WACR.

### Study design

A qualitative design was used to explore the implementation from various stakeholder perspectives. The study consisted of two phases 1) the distribution of the online pre-proforma at the start of the WA Cancer Staging Project, and 2) the distribution of the online post-proforma to stakeholders near the end of the first year of the WA Cancer Staging Project. Various data collection methods were considered (e.g., focus groups and interviews). Due to the short 5-month project timeframe after the project team had been recruited, study approvals were in place, and the project was initially due to finish in June 2022; qualitative proformas (open-ended survey) were chosen as the best method to obtain the data. Research Electronic Data Capture (REDCap) was utilised to host the qualitative proformas online.

### The conceptual framework for the qualitative process evaluation

The Consolidated Framework for Implementation Research (CFIR) was used to guide the qualitative process evaluation. CFIR was chosen because it is a recognised framework for implementing interventions and can be applied to any stage of the evaluation process [29]. It also provides a framework to investigate and assess the implementation of cancer staging, including the barriers and enablers, and a way to organise and communicate findings [29]. CFIR includes five domains with 39 underlying constructs that can potentially influence efforts to change practice and a pragmatic approach to evaluation [30]. See Table 2 for the descriptions of CFIR domains and associated constructs.

**Table 2:** CFIR’s domain descriptions and associated constructs (based on Damschroder et al. [31]). (All five domains and constructs in bold were included in the current study)

1. *Intervention characteristics*: aspects of an intervention that may impact implementation success, including intervention source, evidence strength and quality, relative advantage, **adaptability**, trialability, **complexity,** design quality and packaging, and cost.
2. *Outer setting*: external influences on the intervention implementation, including patient needs and resources, cosmopolitanism, **peer pressure** and external policies and incentives.
3. *Inner setting*: characteristics of the implementing organisation such as structural characteristics, networks and communications, culture, implementation climate, (**tension for change**, **compatibility**, **relative priority**, organisational incentives and rewards, goals and feedback, learning climate), readiness for implementation, **leadership engagement**, **available resources** and access to knowledge and information.
4. *Characteristics of individuals*: individuals’ knowledge and beliefs about the intervention, **self-efficacy**, individual stage of change, individual identification with organisation and other personal attributes that may affect implementation.
5. *Process*: stages of implementation such as planning, engaging, (opinion leaders, formally appointed internal implementation leaders, champions, external change agents), **executing**, and reflecting and evaluating.

CFIR and Participatory Action Research (PAR) are known to complement each other [32, 33]. PAR advocates that those being researched should be partners in the research process, including the research topic, data collection, and analysis and decide what action should happen because of the research findings [34]. Stakeholders participate both as participants and co-researchers and are encouraged to be active in all research and evaluation activities [35, 36]. A PAR approach was used in the project as an implementation strategy [37, 38] to capture multiple voices of stakeholders lived experiences and tacit knowledge through sharing experiences and expertise to guide the implementation and/or target factors influencing implementation. PAR involves a process of self-reflective inquiry where stakeholders undertake to reflect, understand, and improve practices in which they participate and engage [39]. The implementation was designed *with* stakeholders. The co-design process and consultation throughout was conducted over regular meetings with the stakeholders. Stakeholders were involved in the research topic, co-designing the implementation phases (see Fig. 1), decision-making of the implementation progress, possessed the required knowledge and expertise to help improve the implementation, two were involved in the development of the proforma questions, some took part in the proformas, all reviewed the preliminary findings of the current study and were involved in the future directions.

The CFIR was used in the current study to capture the stakeholders’ reflections/perceptions of the implementation. CFIR’s qualitative tool guided the development of qualitative proformas [40]. SS, an experienced qualitative researcher and two stakeholders with cancer staging/registry expertise and population health expertise, consulted on the CFIR questions to include on the proformas. Key evaluative questions were developed by reviewing the CFIR domains and constructs over three meetings to explore which best suited the project aims and balancing the project timeframe and burden on participants. The first meeting covered reviewing CFIR as a framework for guiding the process evaluation and initial review of including/excluding constructs. The second and third meetings continued the reduction of the constructs (See Supplementary Material 1 for the exclusion process).

The number of questions was founded on recommendations for qualitative proformas [38, 41–44]. The pre- and post-proformas comprised 10 questions adapted from the CFIR and one question at the end for the stakeholders to add anything of interest or that had yet to be covered. Proformas have been previously used in rapid qualitative approaches [38, 41–44]. Questions were the same in both proformas to allow comparison but were worded in the present (pre-proforma)/past tense (post-proforma) (See Supplementary Material 2 for the pre-proforma and Supplementary Material 3 for the post-proforma). Final questions were reviewed by an independent and experienced qualitative researcher and also underwent a lay review, with the wording checked for clarity. Minor changes were made based on the feedback.

### Participants

Numerous implementation strategies outlined by Powell et al. [45] were considered for this project and influenced the selection of participants. These included (1) ‘developing an academic partnership’ between the WACR and Curtin University to bring research skills to the implementation, (2) ‘facilitation’ to support the interactive process through recruiting a project team, (3) using an ‘advisory board and workgroups’ and (4) involving ‘consumers’ as stakeholders to oversee and consult on the project and (5) using ‘data experts’ through data scientists to employ the NLP/ML and biostatisticians to demonstrate the value of staged data.

**Fig. 2:**
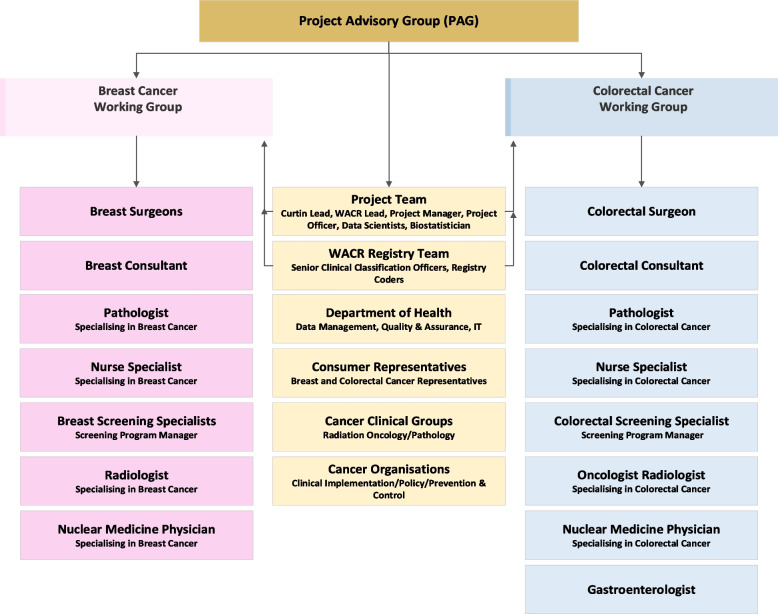
The Project Advisory Group and Working Groups Composition

Purposive sampling was used. The project included a Project Advisory Group (PAG) to provide strategic expert advice and oversight on the project's direction and oversee the expert Breast and Colorectal Cancer Working Groups that consulted on collection queries and the development of the business rules. The study’s inclusion criteria were stakeholders from the PAG and Breast and Colorectal Working Groups. Figure 2 outlines the composition of the PAG and Working Groups.

To enhance anonymity, groups were created for participants to self-classify (see Table 3). This approach has been used in other implementation/qualitative studies [38]. The groups consisted of Clinician, Healthcare staff or Consumer, Registry Staff, or Other. Groups consisted of stakeholders who were seen to hold commonalities [38] or combined to further assist with anonymity. For example, some groups only had one or two members and would be easily identifiable to the researchers and other PAG and Working Group members. The ‘Other’ category was provided as an option for the smaller groups of participants not represented in the named groups and as an option to reduce the number of the various groups (e.g., biostatistician, data management, data scientists, health researchers, etc.) [46]. The groups that participants could self-classify was aimed to assist with anonymity and participation. Consumer representatives received payment for their time.

**Table 3:** Stakeholder characteristics across pre- and post-proformas

**Characteristics**	**Pre-Proforma** **(29/38 completed—76% response rate)** **N (%**^**a**^**)**	**Post-Proforma** **(18/29 completed—62% response rate—18/38 47% overall response rate)** **N (%**^**a**^**)**
Age (years):	(31–76 age range)	(31–76 age range)
30–39	5 (17.2)	4 (22.2)
40–49	7 (24.1)	5 (27.8)
50–59	12 (41.4)	5 (27.8)
60 +	4 (13.8)	3 (16.7)
Not answered	1 (3.5)	1 (5.6)
Role self-classification:		
Clinicians	12 (41.4)	6 (33.3)
Healthcare staff or consumer	6 (20.7)	4 (22.2)
Registry staff	3 (10.3)	2 (11.1)
Other	8 (27.6)	6 (33.3)
Group membership:		
PAG	9 (31.0)	7 (38.9)
Working Group (breast or colorectal)	14 (48.3)	6 (33.3)
Both PAG and Working Group	6 (20.7)	5 (27.8)

^a^Percentages may not equal 100% due to rounding

### Data collection

Stakeholders were invited by email via REDCap to participate in the pre-proforma in March 2022. The email contained the link to the online qualitative pre-proforma. An email reminder was sent via REDCap after no response within one week of the original invite. Consent to participate was provided at the start of the pre-proforma. Participants that took part in the pre-proforma were invited to participate in the post-proforma in May 2022, and the same process for reminding participants was followed. The participant information sheet was present at the start of both proformas to outline the study and what participation involved and acted as a reminder for the post-proforma. The proformas were planned at the beginning of Phase 1 and the end of Phase 5 (see Fig. 1 and Table 1). Due to the coronavirus disease 2019 (COVID-19), delays in obtaining the server to embed the NLP/ML models into the WACR and awaiting the outcome of the project extension funding, the study occurred between Phase 1 and Phase 3 (see Fig. 1 and Table 1).

### Data analysis

Responses were downloaded from REDCap to an Excel file. Proforma responses were anonymised and imported to NVivo [47] to manage the data and aid data analysis. Data were analysed using Ritchie and Spencer’s [48] Framework Analysis to structure and explore the data. Framework analysis was chosen because it is a method developed to address specific questions and can be seen as an applied research approach useful for informing policy and practice [48]. It provided a structured and rigorous process for managing data whilst also allowing for the flexibility associated with qualitative enquiry [49].

Guidelines on conducting a barriers and enablers analysis of a complex tailored intervention have recently been made available by Smith et al. [50]. Smith et al.’s [50] guidelines incorporate CFIR and a novel traffic light labelling grading system using a visual colour coding system for the level of severity of key barriers identified (green for enablers and red for barriers) within Framework Analysis to highlight the levels of positivity and negativity and levels of implementation concern. Smith et al. [50] originally developed this as a methodological roadmap for the Zero Childhood Cancer Precision Medicine Program to identify barriers and enablers. A modified approach to Smith et al. [50] was undertaken for the current study. This included manually coding positive and negative sentiments to capture nuanced responses compared to Smith et al.’s [50] Sentiment Analysis use of automated coding using natural language processing. Sentiment Analysis is often used for analysing big data, e.g. social media [51, 52]. Therefore, manual coding provided an added advantage to remain close and gain an in-depth understanding of the data to better inform the visual presentation of qualitative findings for rapid implementation and interpretation. This study also advanced Smith et al.’s [50] previous work, including pre- and post-proformas for intervention optimisation.

A diverse research team aids to advance the transference of knowledge across disciplines and ensure ethical considerations are considered [53]. The research team involved expertise in cancer staging, cancer nursing, data science, qualitative research, health psychology, implementation science, population-based cancer registries, population health and the WACR. SS, an experienced qualitative researcher and chartered health psychologist with a background in cancer research and implementation science led the analysis. LP (Research Officer with a public health background) was involved in the coding process, adding to the rigour. JS (an Implementation Scientist and qualitative researcher independent of the project with a cancer research background), consulted on the CFIR and adaptions of the grading system for the analysis process. SS undertook reflexive practice by keeping notes that were referred to throughout the iterative analysis process. Regular discussions took place between SS and LP in determining the positive and negative sentiment of statements and SS and JS regarding the CFIR and grading sentiment. In the limited cases, JS also advised on sentiment uncertainty. See Table 4 for the analytical process and description. The diverse stakeholders reviewed preliminary findings. All authors reviewed preliminary and the final findings. The research team composition and the varied group of stakeholders allowed the findings to be reviewed from multiple perspectives.

**Table 4:** Analytical process and description

**Framework Analysis stages**	**Description**
**1. Familiarisation**	Two researchers (SS and LP) immersed in the data independently. This included reading and re-reading the responses of the pre- and post-proformas several times, noting key ideas.
**2. Identifying a thematic framework**	CFIR domains and selected constructs were used as the thematic framework. This was an iterative process and involved revisions. Initially, all 39 CFIR constructs (see Table 2) were reviewed. Refinements were made, and it was agreed after preliminary coding to code to the included 10 constructs (see Table 2) due to the limited responses in the non-included constructs in the proformas. Cross-over between constructs was also noted. Open coding was considered but not used as the researchers found the responses applied to the definitions of CFIR constructs. This was likely as the questions were developed around the CFIR.
**3. Indexing**	Involved applying the framework to the data. Responses were deductively coded in NVivo by each researcher to the included domains and their constructs or moved to another relevant included construct if better suited. This was an iterative comparative process, and the two researchers resolved discrepancies through regular discussions. Coding comparisons were made using coding stripes. Cohen’s Kappa [54] was also conducted to review the levels of agreement between the two researchers. Coder agreement was ‘fair to good’ for the pre-proforma (Kappa = 0.59). Although not required, Kappa was also checked for the post-proformas, and coder agreement had improved to ‘very good’ for the post-proformas (Kappa = 0.86).
**4. Charting**	This step was based on an adapted version of Smith et al. [50] guidelines for determining and grading barriers and enablers. This involved arranging the data into positive and negative statements (barriers and enablers) within each construct. Theme labels were used to capture the essence of the statements for ease of comparison between the two researchers. Short paragraphs or sentences were sometimes separated depending on the positive or negative aspects of the responses. Similarities and differences between the researchers continued through regular meetings. Uncertainty or differences were resolved through the regular discussions. Both researchers provided their opinions before coming to a consensus. In the few cases of uncertainty, JS was consulted on determining sentiment. Results were merged into a matrix, allowing the frequency of positive and negative statements
**5. Mapping and interpretation**	This stage involved SS reviewing patterns in the data and presenting the interpretations. A simplified summary of the constructs was produced based on Smith et al. [50] (see Table 5). Figure 3 provided a visual representation on the change in the negative statements across the constructs from pre-to post proforma among stakeholders. The colour coding in Table 5 and Fig. 3 are based on the traffic light system created by Smith et al. [50] where red is a barrier, orange is barrier/enabler, and green is an enabler. The visual presentation assisted the interpretation of the barriers and enablers of the constructs. Stakeholders reviewed preliminary findings. The interpretation continued in writing the results and was reviewed by all authors. The theme labels assisted in this process and the structure of the positive and negative statements.

### Findings

A total of 38 stakeholders were invited to participate in the process evaluation. Twenty-nine stakeholders participated in the pre-proforma (76% response rate), and 18 participated in the post-proforma (62% response rate/47% overall response rate). All five CFIR domains were included. Key constructs selected within each domain are reported, summarising the barriers and enablers to implementing cancer staging into the WACR, followed by a summary of the findings as outlined in Table 5. Table 5 highlights the data activity event (pre/post-proforma) and the type of response expressed (positive/negative). Table 5 is referred to throughout the findings. All quotes are verbatim.

**Table 5:**
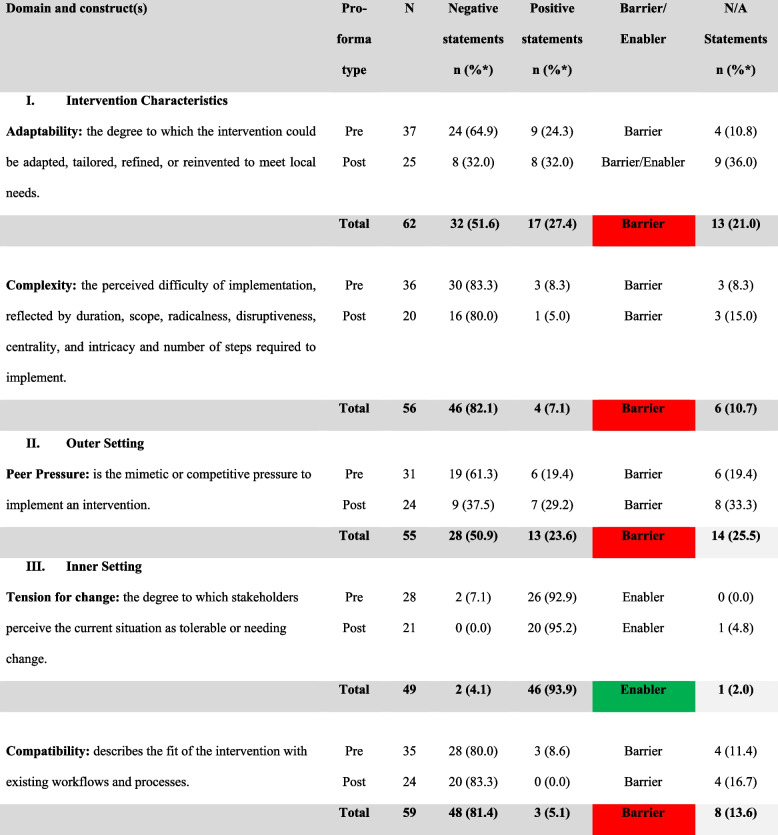

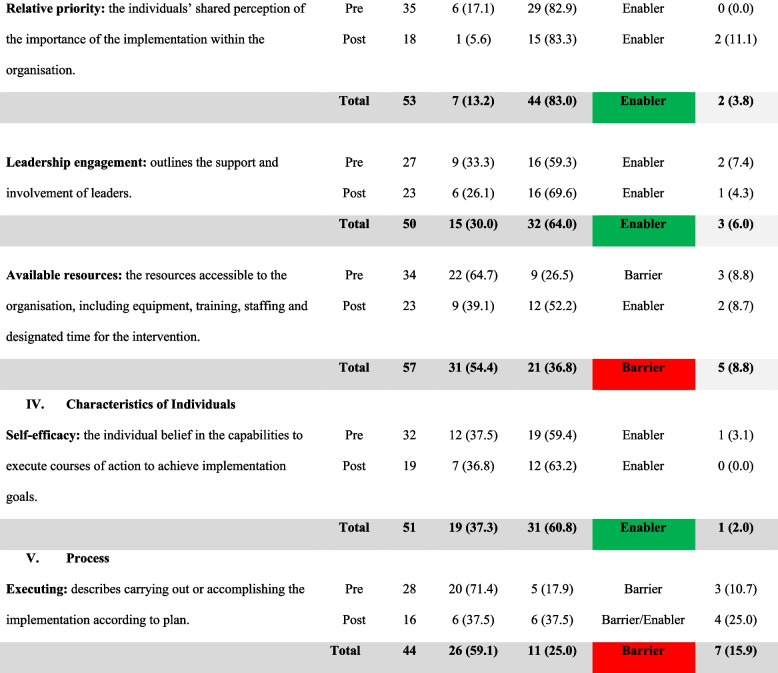
Grading data: a visual presentation of the barriers/enablers of the CFIR constructs

KEY: Traffic light system: Barriers are presented in red = The total negative percentage is higher than the total positive percentage; Enablers are shown in green = The total positive percentage is higher than the total negative percentage; Barrier and Enabler would be presented in orange: The total number of barriers and enablers are equal. *N* = Total number of statements combined across each pre- and post-proforma. *n* = Total number of statements (negative/positive). *N/A* Not Applicable statements

*Percentages may not equal 100% due to rounding

### Domain 1: intervention characteristics

#### Adaptability: overall barrier to implementation

Adaptability is the degree to which current practices can be adapted, tailored, refined, or reinvented [40]. This construct also consists of suggested improvements. More barriers were identified with the pre-proforma. Yet, there was a balance between barriers and enablers in the post-survey, indicating that stakeholders noted possibilities for adapting the intervention with time.

The implementation was described as an ‘expansion of BAU [business as usual]’ *[Healthcare staff/consumer/Pre-Proforma]*. This reflected the existing practices, and the requirements of the WACR (e.g., diagnosis and incidence reporting) will remain the same. The project offered the opportunity for cancer staging at diagnosis to become routinely collected. The current practice of cancer staging in the WACR included being opportunistically and manually captured by the coding team if reported and noted in pathology reports. The project posed rigorous and standardised operational changes through technical adaptions:

Utilisation of modern technologies such as natural language processing and machine learning to extract key pieces of information and build algorithms to determine the stage at diagnosis, ensuring meaningful diagnostic checks are in place to provide context and meaning where stage has been derived *[Registry Staff/Pre-Proforma].*

To enable the implementation, existing databases with various compatibility issues noted (see compatibility construct) needed to be modified for the technical adaptations. If achievable, it was suggested to provide future benefits to the registry coders' workflows:

[Database Name] application changes being assessed to integrate the modelling data into [Database Name] to assist coders in working through their mapping more quicker *[Other/Post-Proforma].*

In turn, the Registry Staff group noted: ‘Coders will need more training on staging’ *[Registry Staff/Post-Survey]* once implemented into the WACR.

Other changes included the ‘integration of all appropriate stakeholders’ *[Clinician/Pre-Proforma]* to consult on the project and business rules and was a positive change for cancer staging to work effectively and attempt to improve the accuracy of data within the WACR by drawing on the varied expertise. Similarly, ‘staffing’ changes *[Clinician/Pre-Proforma]* were also noted as an adaption as the project team had been brought in to assist with the implementation from the project management, data science and epidemiological aspects.

Many participants mentioned personal changes in their practices through the collaboration, with more modifications reported over time from various groups, including knowledge changes, providing expert knowledge, and promoting cancer staging.

Whilst the project was currently working on the registry-derived stage (Tier 2 of the Cancer Staging Tiered Framework) in achieving the best estimate of cancer stage at diagnosis based on the data sources available (see Table 1), recommendations were provided on possible future improvements to achieve accurate data (Tier 1 – complete AJCC collection) (see Table 1). A recurring recommendation was the need for standardised reporting of cancer staging in pathology reports that will provide more accurate information. The standardised reporting was suggested to include ‘TNM and stage in fields that can be extracted…with clear descriptors of whether clinical stage or revised etc.’ *[Clinician/Pre-Proforma]*. Many stakeholders reported concerns that the current system required improvements in terms of data access to provide more accurate staging information. Yet, these concerns were noted as improvements and beyond the current project's scope: ‘We will need to have access to imaging, MDT [multidisciplinary teams], private patients’ data to abstract the relevant information’ *[Registry Staff/Pre-Proforma].* Interestingly, improvements were less frequently reported in the post-survey, perhaps highlighting a focus on the current project over time.

#### Complexity: overall barrier to implementation

Complexity is the perceived difficulty of implementation [40]. Barriers dominated this construct at both time points and centred around the lack of documented examples of cancer registries implementing cancer staging to guide the process. Data gaps complicated the running of the NLP/ML models, and the process was complex to implement. The following quote highlights the change in perception:

Initially I assumed that all the data would be readily accessible from the pathology reports, and it would simply be a case of developing a machine learning/NLP model to capture the information. However, it has been shown to be a lot more complicated as there are significant data gaps plus the pathology reports are not in a consistent format *[Other/Post-Proforma].*

For some, the implementation of cancer staging was described as ‘complex and time consuming and will require considerable consultation’ *[Healthcare staff/consumer/Pre-Proforma]*. Yet, working with stakeholders was highlighted as a benefit to enhance the implementation:

It took a while to understand the pathology reports…We need to [meet] multiple times with the subject matter experts in the working groups/PAG…Once we got the understanding of what to expect of different reports…and different tumour groups…the scope of the…work became much more clear *[Other/Post-Proforma].*

Reviewing two cancer streams highlighted that different considerations needed to be taken onboard for each tumour group:

The pathways are also very different for the two tumour groups...So the rules need to be tumour specific *[Other/Post-Proforma].*

Complexity issues extended to the treatment variations, as not all patients experience the same clinical pathway:

Consideration on how to extract the data for patients that don't have surgery within the 4-month period. Such as rectal cancer patients who have neoadjuvant CRT [chemoradiotherapy], anal scc [squamous cell carcinoma] patients who usually have definitive CRT as well as patients having minimally invasive surgeries *[Healthcare Staff or Consumer/Post-Survey].*

The following further highlights how the patient characteristics and treatment pathways can also impact accurate cancer staging:

A number of cancers will be difficult to stage for clients that do not have active treatment due to old age, comorbidities or personal choice. Reluctant patients who delay diagnosis/staging can be time consuming or may need to be revisited *[Healthcare Staff or Consumer/Pre-Proforma].*

In these instances, patients may be staged clinically, or not at all, and this information may not be received by population-based cancer registries.

Operational barriers were highlighted as complex and included relying on another department's approval:

…the approach is very process heavy requiring change request forms to be created and submitted to a third party [IT department] for review *[Other/Post-Proforma].*

The project experienced delays due to COVID-19 in obtaining the software to integrate cancer staging fully.

### Domain 2: outer setting

#### Peer pressure: overall barrier to implementation

Peer pressure is the mimetic or competitive pressure to implement an intervention [40]. Barriers were more prevalent within this construct, but a reduction in negative responses was noted with the post-proforma.

Incorporating cancer staging into the WACR was suggested to ‘align with international best practice and should be pursued’ *[Healthcare Staff or Consumer/Pre-Proforma]*. Comparisons were made both nationally and internationally, and most stakeholders described that WA was less advanced than other Australian jurisdictions (with repercussions highlighted) and other countries:

WA Cancer Registry is one of the few pop[ulation] registries that does not collect cancer stage at diagnosis - as such it is difficult to compare patient outcomes in WA vs other jurisdictions *[Healthcare Staff or Consumer/Pre-Proforma].*

The SEER database (U.S.) and the U.K. cancer registry data seem to routinely include staging *[Clinican/Pre-Proforma]*

European Network of Cancer Registries have been setting standards and systems for years on staging data etc. *[Healthcare Staff or Consumer/Pre-Proforma].*

It was reported that WA had only conducted cancer staging at the population level with the STaR project in 2015 based on 2011 data, whilst some Australian states had continued. Yet, the current projects approach using NLP/ML was suggested as advanced from other jurisdictions and may explain the reduced number of negative statements with the post-proforma:

WA is [a] fair way behind other jurisdictions when it comes to the collection of staging. However, I'm aware other jurisdictions rely heavily on manual entry *[Registry Staff /Post-Proforma].*

Differences between jurisdictions were suggested highlighting a lack of standardisation.

### Domain 3: inner setting

#### Tension for change: overall enabler to implementation

Tension for change is the degree to which stakeholders perceive the current situation as tolerable or needing change [40], and mainly enablers were noted at both time points.

All responses highlighted that implementing cancer staging into the WACR was a change needed ‘for all cancers’ *[Clinician/Pre-Proforma].* Without it, WA was ‘currently flying blind’ *[Clinician/Pre-Proforma],* suggesting that WA lacks the knowledge and guidance on cancer staging. In support, many stakeholders suggested that having cancer staging information will improve knowledge and understanding in a range of outcomes:

…the addition of some measure of disease stage at diagnosis would add a great deal to our ability to understand differences in survival and patterns of incidence and mortality *[Registry Staff/Pre-Proforma]*.

Access to cancer staging data will aid to ‘benchmark cancer outcomes and determine if WA patients receive treatment as per Optimum Care Pathways’ *[Healthcare Staff or Consumer/Post-Proforma]* that are the national standard of high-quality cancer care that all Australians should experience.

One stakeholder suggested that cancer staging was long overdue: ‘there has been [a need for cancer staging] since before Threlfall's paper in 2005 *[Healthcare Staff or Consumer/Post-Proforma].*

Access to cancer staging data was reported to ‘identify gaps and areas of need’ *[Clinician/Pre-Proforma]* through evaluating early detection, treatments and pathways, health care and screening services:

Staging data could be paired with date of diagnosis which could provide useful information about how well (or not) we are finding certain cancers early *[Other/Pre-Proforma]*.

Without staging information it is very difficult to accurately evaluate the impact of new treatment or diagnostic pathways as they are likely to have a different impact depending on stage. Also stage is important for incidence and prevalence data in order to estimate future health service use *[Other/Post-Proforma]*.

…screening feasibility *[Other/Pre-Proforma]*

Similarly, access to cancer staging data will adhere to recommended guidelines:

The WA Cancer Plan 2020-2025 Implementation Plan subsequently identified the key strategic action to ‘Develop a timely data collection for cancer stage at diagnosis.’ The National Cancer Data Strategy for Australia (2008) recognises population-based registries' lack of nationally standardised Stage of Cancer at Diagnosis Data Collections *[Other/Pre-Proforma]*

One stakeholder highlighted the recent pandemic to be able to make national and international comparisons:

…in the context of COVID worldwide there is a concern that diagnoses were down and…this means more people will be diagnosed at later stages which will affect the QALY [Quality-Adjusted Life Year] cost of COVID - we won’t know this for WA because we won't have the data! *[Healthcare Staff or Consumer/Pre-Proforma]*

#### Compatibility: overall barrier to implementation

Compatibility describes the fit of the intervention with existing workflows and processes [40]. Barriers were primarily identified in this construct. No enablers in the post-proforma were mentioned, suggesting that compatibility was noted as more of an issue over time.

A significant concern was raised around the compatibility of linking the cancer staging process via the NLP/ML models to the current software, which was noted to have existing problems already:

Major issue will be [Database Name] software which is outdated and already cannot cope with the increased amount of histo[pathology] reports coding *[Registry Staff/Pre-Proforma].*

Similarly, it was noted that regular changes to the staging process were needed, and the current process did not enhance compatibility, with a recommendation provided:

…research is very agile and software/database changes may need to be done frequently which does not align well with the strict [Database Name]/[IT Department] processes. A solution would be to have a more isolated/standalone schema for the project *[Other/Post-Proforma].*

The lack of available data sources also hindered the compatibility for accurate cancer staging. Incompatibility was frequently reported around the pathology reporting, often noted as inconsistent across the pathology providers. This lack of standardisation included:

Subtle differences in wording between pathology reports of the different providers *[Clinician/Post-Survey].*

The compatibility of the reporting was also questioned:

…pathology reporting does not really take into account the use of the reports by the WACR *[Other/Post-Survey].*

Some stakeholders reflected on what was and was not compatible during the project:

Missing data can be within the scope of the project, such as missing pathological reports, and beyond the scope of the project, no access to imaging and MDT data *[Registry Staff/Pre-Survey].*

The enablers mentioned in the pre-proforma were limited but included learning from the current project to build a more ‘robust system’ *[Clinician/Pre-Proforma].*

#### Relative priority: overall enabler to implementation

Relative priority is the individuals’ shared perception of the importance of the implementation within the organisation [40]. There was high relative importance from many stakeholders in comparison to barriers. The following are examples of the terms commonly used to describe the importance of implementing cancer staging into the WACR: ‘important,’ ‘worthwhile,’ ‘essential,’ ‘vital,’ and ‘absolutely critical.’ Participants were personally committed to the project, mainly due to the benefits of implementing staging in evaluating outcomes and treatments, survival trends, and supporting research and policy:

To better understand the population groups at risk in WA, to assist in risk adjusting cancer related performance and safety and quality indicators, to support planning and prevention programs and a wide range of research studies, stage at dx [diagnosis] is essential *[Registry Staff/Post-Survey].*

Some stakeholders enjoyed being involved in change processes: ‘It is good to be a part of the information and change to practice/systems’ *[Clinician/Post-Survey].* Yet, some stakeholders noted that the amount of support they could provide for the project will likely be impacted by other competing priorities:

I am relatively committed but relatively busy so will support the process within the limitations of available time *[Clinician/Pre-Survey].*

There was a need to continue promoting the relative importance of cancer staging into the WACR to secure funding for ongoing work for all cancers:

…the greatest issue will be making the case to secure funding for the full implementation of staging for all cancer types *[Healthcare Staff or Consumer/Pre-Survey].*

Cancer staging information will also aid in directing future resources, and the evidence from this project will support this:

We will be able to learn which cancers we need to improve early detection…[Cancer Staging] will be evidence to call upon when arguing for Govt [government] spending e.g. Lung cancer screening *[Other/Pre-Survey].*

#### Leadership engagement: overall enabler to implementation

Leadership engagement outlines the support and involvement of leaders [40]. This construct found more enablers than barriers.

Leadership engagement was noticeable with individuals collaborating with the project and was consistently described as positively influencing implementation. The support and active involvement of the project leaders were essential and frequently mentioned facilitators:

Commitment from the [Project] team yes, commitment from [Funder] yes *[Clinician/Post-Survey]*

The registry team are a dedicated, knowledgeable and committed team *[Healthcare staff/consumer/Pre-Survey]*

The early engagement of the PAG and Working Groups also ensured that other leaders from diverse expertise (e.g., clinicians, consumers, data scientists, biostatisticians, data management, healthcare staff, and health researchers) were also dedicated to the project:

There is a great commitment from working group members as well as PAG *[Other/Post-Survey]*

Yet, although fewer barriers were mentioned within this construct, they are important to highlight. There were concerns that sustainable progress with cancer staging needed to be established with the executive leaders of the health department:

Long term top-down commitment is required, and I see no sign it is there *[Healthcare Staff or Consumer/Pre-Survey].*

…we need more support from the higher levels *[Registry Staff/Pre-Survey].*

There were concerns that cancer staging will not get the priority it deserves due to a lack of understanding of the significance of the data internally from the department and with external bodies (e.g., other clinicians and the broader cancer care community). For example, fear of increased burden of administrative work both internally and externally.

#### Available resources: overall barrier to implementation

Available resources are the resources accessible to the organisation, including equipment, training, staffing and designated time for the intervention [40]. Overall, more barriers were mentioned, but more enablers in comparison to barriers were noted in the post-proforma.

The project was often described as sufficiently resourced and planned due to the funding received*.* The allocated funding enabled the project team to come on board to manage the project, develop the NLP/ML models, and analyse the staging information. Funding had also been covered to purchase the new server to embed the models. The stakeholders' expertise was also suggested as a great resource for the project*.*

Yet, the project was reported as under-resourced ‘in terms of data resources available’ *[Other/Pre-Survey]* in the WACR*.* Sustaining cancer staging and extending the work to include other cancer types was reported to require further funding and sustainable resources. Many described a lack of accountability from the department:

…as with all aspects of the cancer plan, I am concerned the department is not committing sufficient resources in a consistent and sustained fashion. There is a constant worry about funds, rollover of personnel, bids for funding and knock backs *[Healthcare Staff or Consumer/Pre-Survey].*

Additional skilled staff were mentioned as a required resource as cancer staging will still require checks for flagged cases from the NLP/ML models and will add to the current registry workload:

…the registry needs to be funded to ensure they have the skilled staff to collect, enter and clean data in a timely fashion *[Other/Pre-Survey]*

### Domain 4: characteristics of individuals

#### Self-efficacy: overall enabler to implementation

Self-efficacy is the individual belief in the capabilities to execute courses of action to achieve implementation goals [40]. This construct was an enabler of implementation. Collective efficacy was often described rather than self-efficacy, especially from stakeholders not directly involved in the WACR. The efficacy of cancer staging remained consistent over the two-time points. Many participants were confident that cancer staging will occur due to the dedicated team and skilled staff:

Coders are very enthusiastic and keen to incorporate staging and look forward to…the necessary support *[Registry Staff/Pre-Survey]*

One stakeholder revealed that the stakeholder meetings had enhanced their understanding and commitment to addressing the barriers:

Attending the sessions have given me valuable insight into this aspect of reporting and the challenges faced in standardising the data sets…I am committed to helping in anyway [Department Name] can to surface this within [Database Name] *[Other/Pre-Survey]*

The approach used for cancer staging was reported to provide confidence in the transition of practice:

For longevity I think we've taken the right approach by taking a ML/NLP approach. Manual entry whilst achievable always would have been subject to scrutiny and would have introduced manual interpretation and subjective complaints *[Registry Staff/Post-Proforma]*

Yet, for some stakeholders, the delays that had occurred with the project had reduced the level of confidence:

At the beginning and middle of the project, I was highly confident. As the project is nearing conclusion, I am less confident due to issues caused by the procurement and setup of the WACR analytics server as well as dependencies on software development work that would need to be performed by [Department Name] and various process/change requests need to add in the [Database Name] enhancements. *[Other/Post-Survey].*

While some stakeholders were confident that some amount of cancer staging will be implemented, challenges were again focused on the ‘data quality and availability’ *[Other/Pre-Survey]*.

Many stakeholders reported concerns beyond the current project aims based on the time it will take to stage all cancer types: ‘integrating cancer staging for all tumour types will be a long-term initiative *[Other/Pre-Proforma]*. Similarly, the uncertainty of cancer staging happening for all cancer types focused on the lack of resources available and commitment. This caused worry for some: ‘this [cancer staging] is desperately needed information today’ *[Other, Post-Survey].*

### Domain 5: process

#### Executing: overall barrier to implementation

Executing describes carrying out or accomplishing the implementation according to plan [40]. More barriers were identified with the pre-proforma and an equal balance of barriers and enablers for the post-proforma.

Focusing on two tumour streams and the data available was noted by some as beneficial to keep the project in check. Some stakeholders reported that the timeline was realistic. Still, the project was also described as ‘ambitious’ *[Healthcare staff/consumer and Other/Pre-Proforma]*, with more time required for sustaining business as usual. The timeline did not allow for additional troubleshooting for the NLP/ML models or the unexpected delays caused by the differences between the breast and colorectal cancer types and the consultation required from the PAG and working groups:

While this [consultation] has been useful, it…added delays rather than focusing on the core staging extraction components *[Other/Post-Survey]*

The project's goal was to stage two tumour groups with the data available for the best stage estimate. Unfortunately, breast and colorectal cancer staging were not implemented as planned during the process evaluation:

I would say about 75% [to plan]…due to data issues there will be a number of cases that cannot be staged. While the reasons for unstageable have been flagged by the algorithm this will require additional data to be available to resolve *[Other/Post-Survey]*.

### Summary of findings

**Fig. 3:**
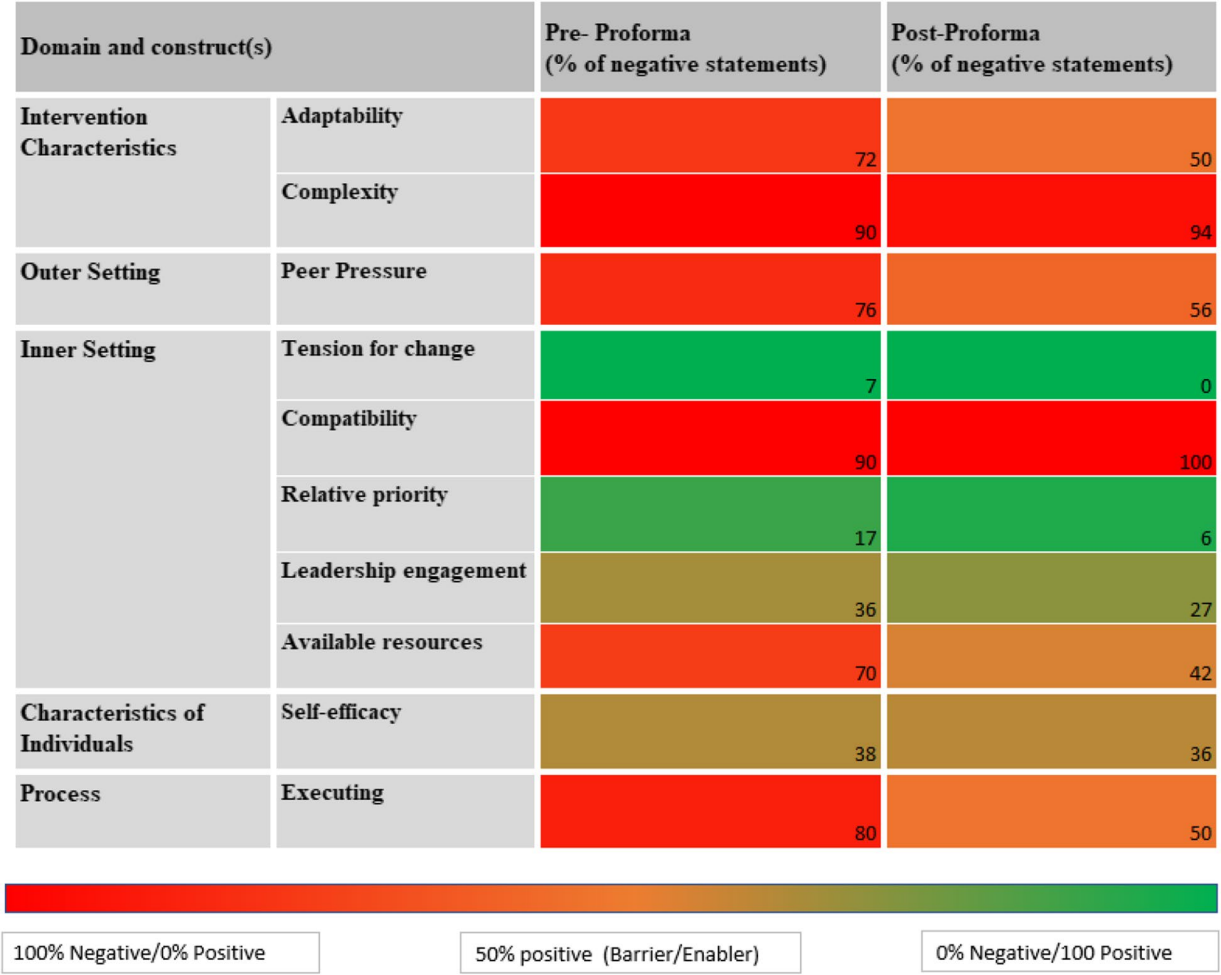
Change in negative statements across the constructs from pre- to post-proforma among stakeholders

In summary, the findings in Table 6 highlight the barriers and enablers listed in order of importance. Figure 3 summarises the change in negative statements across the proformas. Overall, a reduction was noted in negative statements in the post-proforma compared to pre-proforma, with more constructs shifting from barrier (red) to barrier/enabler (orange) or barrier/enabler to enabler (green) (Fig. 3). The highest improvement was from the inner setting domain, with most constructs becoming stronger enablers and available resources becoming an enabler, except for compatibility (Fig. 3). The findings provide an understanding of the barriers/enablers impacting implementation success to be considered within the WA Cancer Staging Project.

**Table 6:** Barriers/Enablers in order of importance

**Barriers**	**Enablers**
1. Complexity (*N* = 56, *n* = 46, 82.1%) 2. Compatibility (*N* = 59, *n* = 48, 81.4%) 3. Executing (*N* = 44, *n* = 26, 59.1%) 4. Available Resources (*N* = 57, *n* = 31, 54.4%) 5. Adaptability (*N* = 62, *n* = 32, 51.6%) 6. Peer Pressure (*N* = 55, *n* = 28, 50.9%)	1. Tension of Change (*N* = 49, *n* = 46, 93.9%) 2. Relative Priority (*N* = 53, *n* = 44, 83.0%) 3. Leadership Engagement (*N* = 50, *n* = 32, 64.0%) 4. Self-efficacy (*N* = 51, *n* = 31, 60.8)

## Discussion

The current study aimed to explore the barriers and enablers of integrating cancer staging into the WACR. This study provides PAR insight into the levels of implementation concern from key stakeholders involved in the WA Cancer Staging Project. Rapid qualitative proformas were used and data were analysed using the well-recognised CFIR and a new novel grading system of implementation concern, qualitatively assessing barriers and enablers. This process evaluation is situated within a project and helps inform the project's next phase. The following summarises the findings, addresses strengths and limitations, and provides recommendations.

Cancer staging was dominantly perceived as necessary. Benefits included improved knowledge and understanding of various outcomes (e.g., survival and incidence patterns, mortality and treatments received by optimum care pathways) and benchmarking nationally and internationally. Implementation barriers included complexity concerns around a lack of standardised pathology reporting for cancer staging from providers, compatibility issues with current systems/workflows, continuous funding, and departmental/higher managerial support.

The findings from the current study highlight that cancer staging is complex, takes considerable time, requires expert consultation, is tumour-specific and requires compatibility checks with existing workflows and processes. Whilst acknowledging that it is vital to have this information to plan for appropriate services, treatments and address inequalities from the perspectives of key stakeholders involved. The findings also provided insight into why staging data is perhaps underreported and lacks standardised routine collection in Australia, as noted previously [14]. Registry-derived stage is a common metric every population-based cancer registry can collect based on their available data and is a helpful starting point to address health inequalities. Whilst the current project is working on the registry-derived stage basis, the findings reveal the strive from key stakeholders to have accurate data, with the gold standard being full AJCC TNM. Recommendations for the completeness of data have been noted previously [55]. Stage data has significant utility at a population level for epidemiological analyses for the planning of services and resource allocation for the predicted demand for cancer-related health services and to enable the evaluation of outcomes from health promotion and screening programs [20]. It is expected that cancer diagnoses are going to continue to increase [5]. Therefore, cancer staging data must be available to assist in preventing cases, improving early diagnosis and reviewing the impact of COVID-19 and possible future crises.

The continued implementation success of the current project is compromised by the lack of sustainable funding and organisational support, and it was highlighted there is a need to have all cancers staged. The project was noted to have positive support from the leaders involved, but department/organisation support was lacking. The findings raise organisational support and perceptions of organisational priority [56], which was perceived as unfavourable to the current implementation outcomes. The project at the time of the study was only funded for 12 months. It was extended till June 2023 and again recently till June 2024 (with an option for a fourth year). The extension has enabled further validation to embed the models and cover the delayed work during COVID-19 in receiving the server. However, this funding is not always guaranteed and has the potential to impact the implementation and planning. Whilst cancer plans and strategies [5, 19, 57] acknowledge the need for cancer staging, sustainable funding must also be considered to ensure recommendations can be implemented appropriately and to the best standard.

The projects use of NLP/ML addresses the WA Cancer Plan 2020–2025 priorities for implementation, identified the key strategic action to ‘Develop a timely data collection for cancer stage at diagnosis’ [5, 19] and is known to assist with manual efforts [14]. In a recent review, it was reported that it is unclear how artificial intelligence tools are currently being used in population-based cancer registries [21]. The current study provides insight from a range of stakeholders, including the expert consultation required to develop the business rules and models to define each stage category and highlights the differences in reporting across pathology providers and data gaps that must be considered.

CFIR is commonly used to facilitate the design, evaluation and implementation of interventions but has also been reported to be burdensome to use, and pragmatic applications have been noted [30]. The current project used CFIR in all stages of the research. In particular, Smith et al.’s [50] approach of using CFIR domains/selected constructs and the grading for implementation concern incorporated within Framework Analysis to identify barriers and enablers offers a transparent and rigorous approach. Smith et al.’s [50] approach provides clear guidance on identifying barriers and enablers. The current study adapted Smith et al.’s [50] original approach to using manual coding and included in-depth and close connection to the data through its rigorous process. The current study also utilised pre- and post-proformas not used previously. Further research is recommended on grading qualitative data of implementation concern comparing Sentiment Analysis used in Smith et al.'s [50] original approach with manual coding as used in the current study.

The current study contributes to qualitative and implementation research. The grading systems visual presentation of the CFIR barriers and enablers and the added changes over time using the traffic light colour coding system captures the summary of qualitative data and levels of implementation concern and provides a rapid way to decipher the barriers and enablers and facilitate understanding amongst stakeholders. In the current study, the visual formats were beneficial in aiding further interpretation of the results (e.g., if barriers/enablers had increased/decreased over time) and provided a clearer understanding of the data in each construct. Researchers, implementation scientists and decision makers have a way of sense-making around immediate project adaptions [50, 58].

### Strengths, limitations and recommendations

Qualitative data were systematically collected and analysed using a rigorous and well-established approach (CFIR), providing an in-depth insight into the stakeholders' views. The PAR approach was beneficial to the implementation bringing guidance to tailoring the project and considerations for its progress. Stakeholders were involved in co-designing the implementation, consulting throughout the project, participating in the current study, consulted on the preliminary findings, directed the action points, needs and implementation concerns of the project, and recommended future research. This study captured multiple stakeholders' perspectives, including registry staff, clinicians, consumers, data scientists, biostatisticians, data management, healthcare staff, and health researchers. Two researchers involved in the analysis were in a neutral position and added to the rigour. The findings have been reviewed with multiple areas of expertise.

Not all the stakeholders took part and there was a further drop in participation with the post-proforma (76% response rate for the pre-proforma and 62% response rate (47% overall response rate) for the post-proforma). This may have been due to the short timeframe of two-months between the pre- and post-proformas. The self-categorisation groups and anonymising responses attempted to reduce identification which may have been a concern for some participants. A recent meta-analysis of online research surveys noted the average response rate is 44.1% [59]. In comparison, the current study’s response rates were positive. Steps taken to improve participation rates included to advise the participants at meetings that the proformas were being emailed, the subject line was concise and included ‘Cancer Staging Project’ to draw attention, emails were personally addressed via REDCap to provide a personalised greeting, the time to complete the proforma as well as the deadline to respond were also provided, as recommended [60]. The stakeholders were very engaged with the project and subject matter which may have also aided the response rates.

Whilst the need to recognise anonymity is important, the group classifications in the current study may be noted as too broad. For example, the ‘Other’ category is not clear on the category of respondents the quotations came from. This was intended for the smaller groups of participants that included one or two members (e.g., biostatisticians, data scientists, data management, health researchers), but may have been used by other participants or used to further protect their identity. Improvements could include using ‘please specify’ as recommended for demographic questions [46] and allows the participants the choice if they want to disclose their group or not. Yet, it is noted that this demographic question did specifically ask participants to: ‘*Please select the option that best describes your role (please select one option).’*

Only selected CFIR constructs were evaluated. Therefore, there may be additional factors relevant to implementing cancer staging into the WACR that were not explored. Additional constructs would have made the proformas longer and may have caused a reduction in response rates. However, CFIR constructs were noted to cross over, and constructs were selected that best suited the study aims and if they covered other constructs. The repetitive construct definitions have been previously noted [30]. Yet, CFIR enables future comparisons with other cancer staging process evaluations and other interventions through its standardised constructs [61].

Standardising the proforma questions was used for ease of comparison. However, some questions may have been more applicable to stakeholders directly involved in the staging process and may explain the ‘not applicable’ responses. Future work may target proforma questions to each stakeholder group.

Some barriers and enablers may not be identified due to the project not being fully implemented at the time of the post-proforma. Similarities in responses were noted between the pre- and post-proformas responses due to the short timeframe. The data was therefore treated as a whole, and differences, when possible, between the two timeframes, were highlighted.

### Implications

The findings of the current study have various implications. From a policy aspect, timely cancer staging data is a priority within WA [19], and nationally it is recognised that there is a lack of standardised cancer staging within population-based cancer registries [14]. A new National Cancer Plan is expected in late 2023 with collection of staging expected to be a continued focus. As health systems strive for equitable cancer care, policies, prevention, and detection to optimise early diagnosis of cancer will remain critical. National cancer stage collection will aid to ensure national strategies can target the most vulnerable. Obtaining this data has implications on population health and with clinicians and patients enabling analysis in trends and potential causal factors, changes in incidence and mortality over time [6], evaluating health inequalities [12] and practice implications regarding access to and the impacts of cancer-related healthcare interventions, screening and cancer services [6]. There is varied collection of staging data across Australia and the findings will aid to inform the complexities of staging collection and the Cancer Staging Tiered Framework developed within the implementation can assist national discussions on standardising cancer staging. This study captures an approach of implementing cancer staging into a population-based cancer registry in WA which other population-based registries considering this data collection can learn from and/or adopt. The current study tapped into the PAR approach used in the WA Cancer Staging Project through the CFIR and provided a reflection of the process of implementing cancer staging into the WACR. Incorporating the knowledge of the levels of implementation concern will enable future work to develop implementation strategies to ameliorate barriers and support enablers early on. It also targets needs, potential extensions of the project reviewed with the stakeholders. The following areas of future development include:

- ***Addressing data gaps:*** Access to imaging, MDT software or standardised reporting on pathology notifications would provide more accurate cancer staging data. The first recommendation will be to discuss the project's findings, highlight the inconsistencies with pathology providers, and explore standardising cancer staging reporting in pathology reports.
- ***Standalone database schema for cancer staging:*** This would prevent the labour-intensive approvals required from IT departments and overloading existing work processes.
- ***Rapid-cycle evaluation:*** The visual grading system method with levels of implementation concern should be conducted at regular intervals, such that the rapid evaluation could be ongoing using rapid-cycles at different time points to evaluate the implementation process in a timely manner and learn about adaption as change occurs to help predict implementation success. This will provide further insights when the data is triangulated against the evidence on the influence of barriers and enablers.
- ***Additional cancer streams:*** Expansion of cancer staging using NLP/ML for additional common tumour groups.
- ***Standardising cancer staging:*** Contribute and lead national standardised collection discussions. The addition of staging data in the WACR output dataset would facilitate use locally, nationally and for research. By promoting the implementation progress of the WA Cancer Staging Project and the Cancer Staging Tiered Framework as an approach for standardisation may assist other registries within Australia and internationally to collect cancer staging data and enable cancer staging comparisons that are currently lacking.

Following on from the process evaluation and through consultation with the stakeholders, the WA Cancer Staging Project is currently working on the data gaps with pathology providers, further cancer streams and are in talks nationally regarding standardising cancer staging.

## Conclusion

The findings highlight the levels of implementation concern from stakeholders, including those that hinder and aid the integration. These findings will aid further review of the implementation, including data gaps and future sustainability and could be a valuable guide to other population-based cancer registries nationally and internationally. The study also provides an adapted rapid qualitative approach to evaluating complex interventions and establishing barriers and enablers. Introducing the traffic light system to explore implementation concerns is a novel approach that recognises the need to evolve CFIR to display qualitative data in visual formats that can be rapidly interpreted by stakeholders. Further research is needed to better understand the use of visualising qualitative data and the continued importance of its application to decision-making across diverse settings and contexts in implementation research.

## Supplementary Information

**Additional file 1: Supplementary 1.** Applying CFIR constructs to the WA Cancer Staging Project. **Supplementary 2.** Pre-Proforma Questions. **Supplementary 3.** Post-Proforma Questions.

## Data Availability

The datasets generated and analysed during the current study are available from the corresponding author on reasonable request. The data are not publicly available due to containing information that could compromise research participant privacy/consent.

## Abbreviations

AJCC: American Joint Committee for Cancer
CFIR: Consolidated Framework for Implementation Research
COVID-19: Coronavirus disease 2019
MDT: Multidisciplinary Team
ML: Machine Learning
NLP: Natural Language Processing
PAG: Project Advisory Group
PAR: Participatory Action Research
REDCap: Research Electronic Data Capture
STaR: Stage, Treatment and Recurrence
TNM: Tumour, Node, Metastasis
UK: United Kingdom
US: United States
WA: Western Australia
WACR: Western Australian Cancer Registry
