# Supplementary material for: “Currently flying blind” Stakeholders’ perceptions of implementing statewide population-based cancer staging at diagnosis into the Western Australian Cancer Registry: a rapid qualitative process evaluation of the WA Cancer Staging Project
Source: BMC Health Serv Res. 2023 Jul 15;23:758. doi: 10.1186/s12913-023-09662-7 (PMC10349432; doi:10.1186/s12913-023-09662-7)
Supplement: Supplementary file 1 — Additional file 1: Supplementary 1. Applying CFIR constructs to the WA Cancer Staging Project. Supplementary 2. Pre-Proforma Questions. Supplementary 3. Post-Proforma Questions. [file 12913_2023_9662_MOESM1_ESM.docx]

**Supplementary 1: Applying CFIR constructs to the WA Cancer Staging Project**

| 1. **INTERVENTION CHARACTERISTICS** | **Short description of construct** | **Round 1** | **Round 2** | **Round 3** |
| --- | --- | --- | --- | --- |
| Intervention Source | Perception of key stakeholders about whether the intervention is externally or internally developed. | **X** | **X** | **X** |
| Evidence Strength & Quality | Stakeholders’ perceptions of the quality and validity of evidence supporting the belief that the intervention will have desired outcomes. | **✓** | **X** | **X** |
| Relative Advantage | Stakeholders’ perception of the advantage of implementing the intervention versus an alternative solution. | **✓** | **X** | **X** |
| Adaptability | The degree to which an intervention can be adapted, tailored, refined, or reinvented to meet local needs. | **✓** | **✓** | **✓** |
| Trialability | The ability to test the intervention on a small scale in the organisation, and to be able to reverse course (undo implementation) if warranted. | **✓** | **X** | **X** |
| Complexity | Perceived difficulty of implementation, reflected by duration, scope, radicalness, disruptiveness, centrality, and intricacy and number of steps required to implement. | **✓** | **✓** | **✓** |
| Design Quality & Packaging | Perceived excellence in how the intervention is bundled, presented, and assembled. | **X** | **X** | **X** |
| Cost | Costs of the intervention and costs associated with implementing the intervention including investment, supply, and opportunity costs. | **X** | **X** | **X** |
| 1. **OUTER SETTING** |  |  |  |  |
| Patient Needs & Resources | The extent to which patient needs, as well as barriers and facilitators to meet those needs, are accurately known and prioritised by the organisation. | **X** | **✓** | **X** |
| Cosmopolitanism | The degree to which an organisation is networked with other external organisations. | **X** | **X** | **X** |
| Peer Pressure | Mimetic or competitive pressure to implement an intervention; typically because most or other key peer or competing organisations have already implemented or are in a bid for a competitive edge. | **✓** | **✓** | **✓** |
| External Policy & Incentives | A broad construct that includes external strategies to spread interventions, including policy and regulations (governmental or other central entity), external mandates, recommendations and guidelines, pay-for-performance, collaboratives, and public or benchmark reporting. | **✓** | **X** | **X** |
| 1. **INNER SETTING** |  |  |  |  |
| Structural Characteristics | The social architecture, age, maturity, and size of an organisation. | **X** | **X** | **X** |
| Networks & Communications | The nature and quality of webs of social networks and the nature and quality of formal and informal communications within an organisation. | **X** | **X** | **X** |
| Culture | Norms, values, and basic assumptions of a given organisation. | **X** | **X** | **X** |
| Implementation Climate | The absorptive capacity for change, shared receptivity of involved individuals to an intervention, and the extent to which use of that intervention will be rewarded, supported, and expected within their organisation. | **✓** | **X** | **X** |
| Tension for Change | The degree to which stakeholders perceive the current situation as intolerable or needing change. | **✓** | **✓** | **✓** |
| Compatibility | The degree of tangible fit between meaning and values attached to the intervention by involved individuals, how those align with individuals’ own norms, values, and perceived risks and needs, and how the intervention fits with existing workflows and systems. | **✓** | **✓** | **✓** |
| Relative Priority | Individuals’ shared perception of the importance of the implementation within the organisation. | **✓** | **✓** | **✓** |
| Organisational Incentives & Rewards | Extrinsic incentives such as goal-sharing awards, performance reviews, promotions, and raises in salary, and less tangible incentives such as increased stature or respect. | **✓** | **X** | **X** |
| Goals and Feedback | The degree to which goals are clearly communicated, acted upon, and fed back to staff, and alignment of that feedback with goals. | **X** | **X** | **X** |
| Learning Climate | A climate in which: a) leaders express their own fallibility and need for team members’ assistance and input; b) team members feel that they are essential, valued, and knowledgeable partners in the change process; c) individuals feel psychologically safe to try new methods; and d) there is sufficient time and space for reflective thinking and evaluation. | **X** | **X** | **X** |
| Readiness for Implementation | Tangible and immediate indicators of organisational commitment to its decision to implement an intervention. | **✓** | **X** | **X** |
| Leadership Engagement | Commitment, involvement, and accountability of leaders and managers with the implementation. | **✓** | **✓** | **✓** |
| Available Resources | The level of resources dedicated for implementation and ongoing operations, including money, training, education, physical space, and time. | **✓** | **✓** | **✓** |
| Access to Knowledge & Information | Ease of access to digestible information and knowledge about the intervention and how to incorporate it into work tasks. | **✓** | **✓** | **X** |
| 1. **CHARACTERISTICS OF INDIVIDUALS** |  |  |  |  |
| Knowledge & Beliefs about the Intervention | Individuals’ attitudes toward and value placed on the intervention as well as familiarity with facts, truths, and principles related to the intervention. | **✓** | **X** | **X** |
| Self-efficacy | Individual belief in their own capabilities to execute courses of action to achieve implementation goals. | **✓** | **✓** | **✓** |
| Individual Stage of Change | Characterisation of the phase an individual is in, as he or she progresses toward skilled, enthusiastic, and sustained use of the intervention. | **X** | **X** | **X** |
| Individual Identification with Organisation | A broad construct related to how individuals perceive the organisation, and their relationship and degree of commitment with that organisation. | **X** | **X** | **X** |
| Other Personal Attributes | A broad construct to include other personal traits such as tolerance of ambiguity, intellectual ability, motivation, values, competence, capacity, and learning style. | **X** | **X** | **X** |
| 1. **PROCESS** |  |  |  |  |
| Planning | The degree to which a scheme or method of behaviour and tasks for implementing an intervention are developed in advance, and the quality of those schemes or methods. | **✓** | **X** | **X** |
| Engaging | Attracting and involving appropriate individuals in the implementation and use of the intervention through a combined strategy of social marketing, education, role modelling, training, and other similar activities. | **✓** | **X** | **X** |
| Opinion Leaders | Individuals in an organisation who have formal or informal influence on the attitudes and beliefs of their colleagues with respect to implementing the intervention. | **X** | **X** | **X** |
| Formally Appointed Internal Implementation Leaders | Individuals from within the organisation who have been formally appointed with responsibility for implementing an intervention as coordinator, project manager, team leader, or other similar role. | **X** | **X** | **X** |
| Champions | “Individuals who dedicate themselves to supporting, marketing, and ‘driving through’ an [implementation]” [101] (p. 182), overcoming indifference or resistance that the intervention may provoke in an organisation. | **✓** | **X** | **X** |
| External Change Agents | Individuals who are affiliated with an outside entity who formally influence or facilitate intervention decisions in a desirable direction. | **X** | **X** | **X** |
| Executing | Carrying out or accomplishing the implementation according to plan. | **✓** | **✓** | **✓** |
| Reflecting & Evaluating | Quantitative and qualitative feedback about the progress and quality of implementation accompanied with regular personal and team debriefing about progress and experience. | **✓** | **X** | **X** |
| **Number of constructs** | **Total (39)** | **23** | **12** | **10** |

**Supplementary 2: Pre-Proforma Questions**

**PART 1 – Demographics:**

1. **What is your age?**
2. **What is your gender?** Please identify the gender that you most identify with.

ð Male

ð Female

ð Unspecified/Other

ð Prefer not to say

1. **Please select the option that best describes your role (please select one option).**

ð Registry staff

ð Clinician

ð Healthcare staff or consumer

ð Other

1. **Please select the option that best describes your membership to the Cancer Staging Project.**

ð Project Advisory Group

ð Working Group

ð Both

**PART 2 – Survey questions:**

1. **Is there a strong need for cancer staging within the cancer registry?**

*Please detail your response.*

1. **Please describe the kinds of changes or alterations you think you will need to make for the cancer staging integration to work effectively in the cancer registry.**

*Please consider if you think you will be able to make those changes.*

1. **Please describe your thoughts on the complexity of integrating cancer staging into the cancer registry.**

*Please consider the following aspects: duration, scope, intricacy and whether integrating cancer staging reflects a clear departure from existing practices.*

1. **Please describe your understanding of how the WA Cancer Registry compares to other cancer registry jurisdictions (including and not limited to Australia)?**
2. **Please describe the likely issues or complications that you feel may arise in integrating cancer staging into the cancer registry?**
3. **Please describe how important it is to you to implement the integration of cancer staging into the cancer registry?**
4. **How would you describe your current feelings on the commitment or support implementing cancer staging into the cancer registry?**
5. **Do you feel the current plan has sufficient resources dedicated to integrating cancer staging into the cancer registry?**

*Please detail your response*

1. **How confident are you that cancer staging will be successfully implemented into the cancer registry?**

*Please provide detail in your answer.*

1. **Do you think that the proposed timeline is realistic?**

*Please provide detail in your answer.*

1. **If you have any other comments or questions about the integration of cancer staging into the cancer registry, please use the space below.**

*Please provide detail in your answer.*

**Supplementary 3: Post-Proforma Questions**

**PART 1 – Demographics:**

1. **What is your age?**
2. **What is your gender?** Please identify the gender that you most identify with.

ð Male

ð Female

ð Unspecified/Other

ð Prefer not to say

1. **Please select the option that best describes your role (please select one option).**

ð Registry staff

ð Clinicians

ð Healthcare staff or consumer

ð Other

1. **Please select the option that best describes your membership to the Cancer Staging Project.**

ð Project Advisory Group

ð Working Group

ð Both

**PART 2 – Survey questions:**

1. **Was there a strong need for cancer staging within the cancer registry?**

*Please detail your response.*

1. **Please describe the changes or alterations you had to make for the cancer staging integration to work effectively in the cancer registry.**

*Please consider what worked well and what did not.*

1. **Please describe your thoughts on how complex integrating cancer staging into the cancer registry was.**

*Please consider the following aspects: duration, scope, intricacy and whether integrating cancer staging reflects a clear departure from existing practices.*

1. **Please describe your understanding of how the WA Cancer Registry compares to other cancer registry jurisdictions (including and not limited to Australia)?**
2. **Please describe the issues or complications that arose in integrating cancer staging into the cancer registry?**
3. **Please describe how important it is to you to implement the integration of cancer staging into the cancer registry?**
4. **Do you feel there has been sufficient commitment or support implementing cancer staging into the cancer registry?**
5. **Do you feel there was sufficient resources dedicated to integrating cancer staging into the cancer registry?**
6. **How confident are you that cancer staging will transition into business as usual?**

*Please provide detail in your answer.*

1. **Has the integration of cancer staging into the cancer registry been implemented according to plan?**

*Please provide detail in your answer*

1. **If you have any other comments or questions about the integration of cancer staging into the cancer registry, please use the space below**

*Please provide detail in your answer.*
